# The *Tg*(*ccnb1:EGFP*) transgenic zebrafish line labels proliferating cells during retinal development and regeneration

**Published:** 2008-05-19

**Authors:** Sean C. Kassen, Ryan Thummel, Christopher T. Burket, Laura A. Campochiaro, Molly J. Harding, David R. Hyde

**Affiliations:** Department of Biological Sciences and Center for Zebrafish Research, University of Notre Dame, Notre Dame, IN

## Abstract

**Purpose:**

To create the *Tg*(*ccnb1:EGFP*)^nt18^ zebrafish line that spatially and temporally labels retinal progenitor cells with enhanced green fluorescent protein (EGFP) during zebrafish retinal development and regeneration.

**Methods:**

We cloned the 1.5 kb promoter region of the zebrafish *cyclin B1* (*ccnb1*) gene upstream of the *EGFP* gene in the Tol2 vector, which was used to generate the stable *Tg*(*ccnb1:EGFP*)^nt18^ transgenic zebrafish line. Immunohistochemistry and in situ hybridization techniques verified that the *ccnb1:EGFP* transgene was expressed in retinal progenitor cells during retinal development, in the undamaged adult retina, and in the regenerating adult retina.

**Results:**

At 36 h post-fertilization, both the enhanced green fluorescent protein (EGFP) and proliferating cell nuclear antigen (PCNA) expressions were observed throughout the developing transgenic retina, but they became restricted to the circumferential marginal zone by five days post-fertilization. In situ hybridization confirmed that this EGFP expression matched the *cyclin B1* mRNA expression pattern. In comparison to the *Tg(1016a1tubulin:EGFP)* transgenic line that expresses EGFP in neuronal progenitor cells, the *Tg*(*ccnb1:EGFP*)^nt18^ line more faithfully follows the rise and fall of PCNA expression through the developing retina and brain. In the adult retina, there are three cell types that continue to proliferate, the Müller glia in the inner nuclear layer, the rod precursor cells in the outer nuclear layer, and the stem cells in the circumferential marginal zone. In the *Tg*(*ccnb1:EGFP*)^nt18^ retina, EGFP coexpressed with PCNA in all three of these proliferating cell types. Exposing the adult retina to constant intense light destroys the rod and cone photoreceptors and induces an increase in the number of proliferating Müller glia, which produces actively dividing neuronal progenitor cells that migrate to the outer nuclear layer (ONL) and replenish the lost photoreceptors. Following constant light damage, *Tg*(*ccnb1:EGFP*)^nt18^ zebrafish expressed EGFP in both the proliferating Müller glia and the migrating neuronal progenitor cells.

**Conclusions:**

The spatial and temporal patterning of EGFP expression in the *Tg*(*ccnb1:EGFP*)^nt18^ line directly reflects the known locations of proliferating cells in the zebrafish retina, making it a useful marker to study the transient nature of neuronal progenitor cells during the development and regeneration of the zebrafish retina.

## Introduction

Over the past 20 years, the zebrafish has developed into an exceptional model system to study early neurogenesis due to its rapid development and because the transparent embryos allow easy visualization of the neurons [1]. The sequence of the zebrafish genome is almost completed, Cre/lox strategies are being developed with inducible transgenes [2-4], forward genetic screens isolated important and interesting developmental mutants [5-8], and morpholinos can specifically knockdown the expression of critical proteins during eye development [9-12]. The use of transgenics to express fluorescent markers in the transparent embryos and adults is an effective approach to monitor gene expression and cell behavior [13,14]. The zebrafish has also become an important model to study regeneration of a variety of tissues including the heart [15], fins [16,17], and retina [18-22]. Several different damage models demonstrated that all the neuronal classes in the retina can be regenerated [19,21-26].

The zebrafish retina arises from a sheet of undifferentiated neuroepithelial progenitor cells [27,28], which yields a functional retina three days post-fertilization [29]. This rapid development makes zebrafish a useful model for understanding retinal development as well as retinal diseases [1,27,30,31]. Large-scale mutagenesis screens to identify mutants that disrupt zebrafish eye and retinal development [7,27,28,32,33], and the generation of an assortment of transgenic lines [14,34], has revealed and will continue to uncover important molecules and pathways in the development, maintenance, and function of the vertebrate retina.

After development, there are two areas of persistent neurogenesis in the adult zebrafish retina, the circumferential marginal zone (CMZ) and dedifferentiating Müller glia [18,20,22,23,35]. The CMZ is the source of all retinal cell types in the growing adult retina except rod photoreceptors. The rod photoreceptor lineage begins with proliferating Müller glia [36], which generate neuronal progenitor cells that migrate to the outer nuclear layer (ONL) where they become rod precursor cells that are committed to differentiating into rod photoreceptors [35]. The proliferation of Müller glia to generate neuronal progenitors is analogous to the well established role of the radial glia in mammalian brain neurogenesis [37-39]. Recently, Müller glia in the mammalian retina were also shown to possess a proliferative ability in vivo [40], and the capacity to produce several retinal cell types including photoreceptors [41].

Upon damage to the zebrafish retina, the Müller glia reenter the cell cycle and produce multipotent neuronal progenitors that can replenish all cell types of the retina [19,22,23,26,36]. Many different damage paradigms exist that elicit a regenerative response, including laser injury [25], injection of neurotoxins [24,42], surgical removal of the retina [26], localized heat [23], retinal puncture [22], and constant light damage [21,43]. We extensively characterized the cellular and molecular mechanisms involved in light-induced retinal degeneration and regeneration [19,21,44]. Constant intense light exposure to the adult zebrafish retina destroys rod and cone photoreceptors [21]. Subsequent to the retinal damage, the Müller glia proliferate and give rise to neuronal progenitors that will replenish the lost photoreceptors [21]. After constant light damage, histological and immunological analysis revealed the cellular stages of photoreceptor degeneration and their subsequent regeneration [19]. A gene microarray analysis was performed to find potential gene candidates that might play a role during zebrafish retinal regeneration [19]. However, there still remains a limited number of retinal progenitor markers that would allow for better characterization of the cellular events and various cell lineages that arise from these cell types during retinal regeneration. Currently, there are two transgenic lines that express enhanced green fluorescent protein (EGFP) in retinal neuronal progenitor cells, *Tg(1016α1tubulin:EGFP)* [22] and *Tg*(*olig2:EGFP*)*^vu12^* [45]. While the *1016α1tubulin:EGFP* transgene appears to accurately express EGFP in proliferating neuronal progenitor cells, the neuronal progenitors begin expressing PCNA much earlier than EGFP in the ouabain-damaged *Tg*(*olig2:EGFP*)*^vu12^* retina [24]. This suggests that these two transgenes label different populations of neuronal progenitor cells. A third transgenic line, *Tg*(*vsx2:GFP*)*^nns1^*, uses the zebrafish *chx10* promoter to drive EGFP expression [46]. This promoter likely expresses green fluorescent protein (GFP) in neuronal progenitors because the *vsx2* transcript has been localized to the neuronal progenitors in the light-damaged retina [23]. Proliferating neuronal progenitors can also be identified by in situ hybridization to the *nestin* mRNA [47], expression of proliferating cell nuclear antigen (PCNA), which labels the nuclei of dividing cells, the incorporation of BrdU into replicating DNA, or the immunolabeling of Pax6 [43]. However, these markers can only be visualized after retinal tissue is fixed and processed.

To label and characterize the proliferating neuronal progenitor cell populations during retinal development and regeneration, we cloned the 1.5 kb promoter region of the *cyclin B1* (*ccnb1*) gene upstream of the *EGFP* gene to generate the transgenic zebrafish line, *Tg*(*ccnb1:EGFP*)^nt18^. Cyclin B1 is a member of the cyclin family of proteins and regulates the transition from G_2_ to mitosis of the cell cycle [48,49]. The microarray and quantitative real time polymerase chain reaction (PCR) experiments performed on different damage models of the zebrafish retina confirmed that *cyclin B1* mRNA is significantly upregulated during the proliferative stages of retinal regeneration [19,24]. Thus, the *cyclin B1* is a good candidate to regulate the expression of a marker in retinal progenitor cells during retinal degeneration and regeneration.

**Figure 1:**
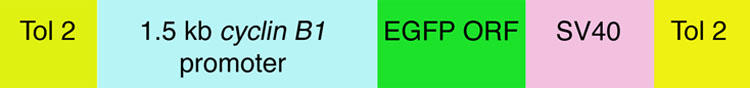
Diagram of the *Tg*(*ccnb1:EGFP*)^nt18^ expression vector. The pT2KXIG plasmid containing the 1.5 kb *cyclin B1* promoter (light blue) was cloned upstream of the enhanced green fluorescent protein (EGFP) open reading frame (ORF; green), which was located within the Tol2 transposable element (yellow) in the pT2KXIG plasmid. The SV40 transcriptional termination and polyA signal (pink) follows downstream of the open reading frame.

Here, we describe the successful generation of the *Tg*(*ccnb1:EGFP*)^nt18^ zebrafish line. During retinal development, PCNA and EGFP are expressed in retinal progenitor cells at 36 h post-fertilization (hpf) in the *Tg*(*ccnb1:EGFP*)^nt18^ line. In situ hybridization confirmed that the *cyclin B1* antisense RNA probe corresponded with EGFP expression in the *Tg*(*ccnb1:EGFP*)^nt18^ line at 48 hpf. At this time, cells in the central retina have exited the cell cycle and terminated both PCNA and EGFP expression in the *Tg*(*ccnb1:EGFP*)^nt18^ line. At 72 and 120 hpf, PCNA and EGFP expression persists near the retinal margin, which corresponds to the circumferential marginal zone in the adult retina. This differed from the *1016α1tubulin:EGFP* transgene, which was expressed throughout the central retina at 48 and 72 hpf. In the undamaged adult *Tg*(*ccnb1:EGFP*)^nt18^ retina, EGFP is expressed in all three known areas of retinal proliferation: the CMZ, inner nuclear layer (INL) Müller glia, and outer nuclear layer (ONL) rod precursor cells. Finally, in the light-damaged *Tg*(*ccnb1:EGFP*)^nt18^ retina, EGFP is expressed in the proliferating Müller glia, the neuronal progenitors, and rod precursor cells. Therefore, the spatial and temporal EGFP expression pattern in the *Tg*(*ccnb1:EGFP*)^nt18^ retina directly reflects known locations of cell proliferation in the zebrafish retina.

## Methods

### Zebrafish maintenance and light lesion protocol

Adult zebrafish (*Danio rerio*) were raised under normal facility lights (300 lux; 14 h light:10 h dark) in the Center for Zebrafish Research at the University of Notre Dame according to established protocols. Adult *albino* zebrafish were dark-treated for 14 days and then subjected to constant intense light (18,000 lux) using four halogen lamps [19]. Retinas were examined at 0 h, 48 h, and 72 h of light exposure. Control animals (0 h intense light exposure) of the same age and genotype were raised under normal facility light conditions. The adult transgenic fish used for immunohistochemical analyses were seven to nine months post-fertilization. Prior to enucleation, fish were deeply anesthetized in 0.2% 2-phenoxyethanol and euthanized by anesthetic overdose. All experimental protocols were approved by the animal use committee at the University of Notre Dame and were in compliance with the US Public Health Service and ARVO statement for the use of animals in vision research.

### Transgenic line generation

A 1.5 kb region of the zebrafish *cyclin B1* promoter was PCR-amplified with primers containing an XhoI restriction site on the forward primer and BamHI restriction sequence on the reverse primer (Forward: 5′-CTC GAG CCT TAA CTG AAG ACC GCA CCT GC-3′; Reverse: 5′-GGA TCC CTG CTT TCT TAG TTT AGA GTA GG-3′). The product was cloned upstream of the *EGFP* reporter gene in a plasmid containing the zebrafish Tol2 transposable element (pT2KXIG) [50]. The plasmid was purified using the Qiagen Maxi Prep Kit (Qiagen, Valencia, CA) and extracted by phenol/chloroform. This construct along with in vitro transcribed Tol2 transposase mRNA was injected into wild-type (AB strain) embryos [2] that were between one and four cells. The EGFP-positive F0 fish were outcrossed to AB fish. The F1 generation carriers were identified by EGFP expression and used to generate stable and independent *cyclin B1:EGFP* transgenic lines. Two stable lines were examined and found to generate indistinguishable EGFP expression patterns in the developing and adult retina. One of these lines, *Tg*(*ccnb1:EGFP*)^nt18^, yielded ~50% EGFP-positive progeny when mated to AB fish, which suggests it has a single Tol2 insertion. This *Tg*(*ccnb1:EGFP*)^nt18^ line was used in all of the data presented.

**Figure 2:**
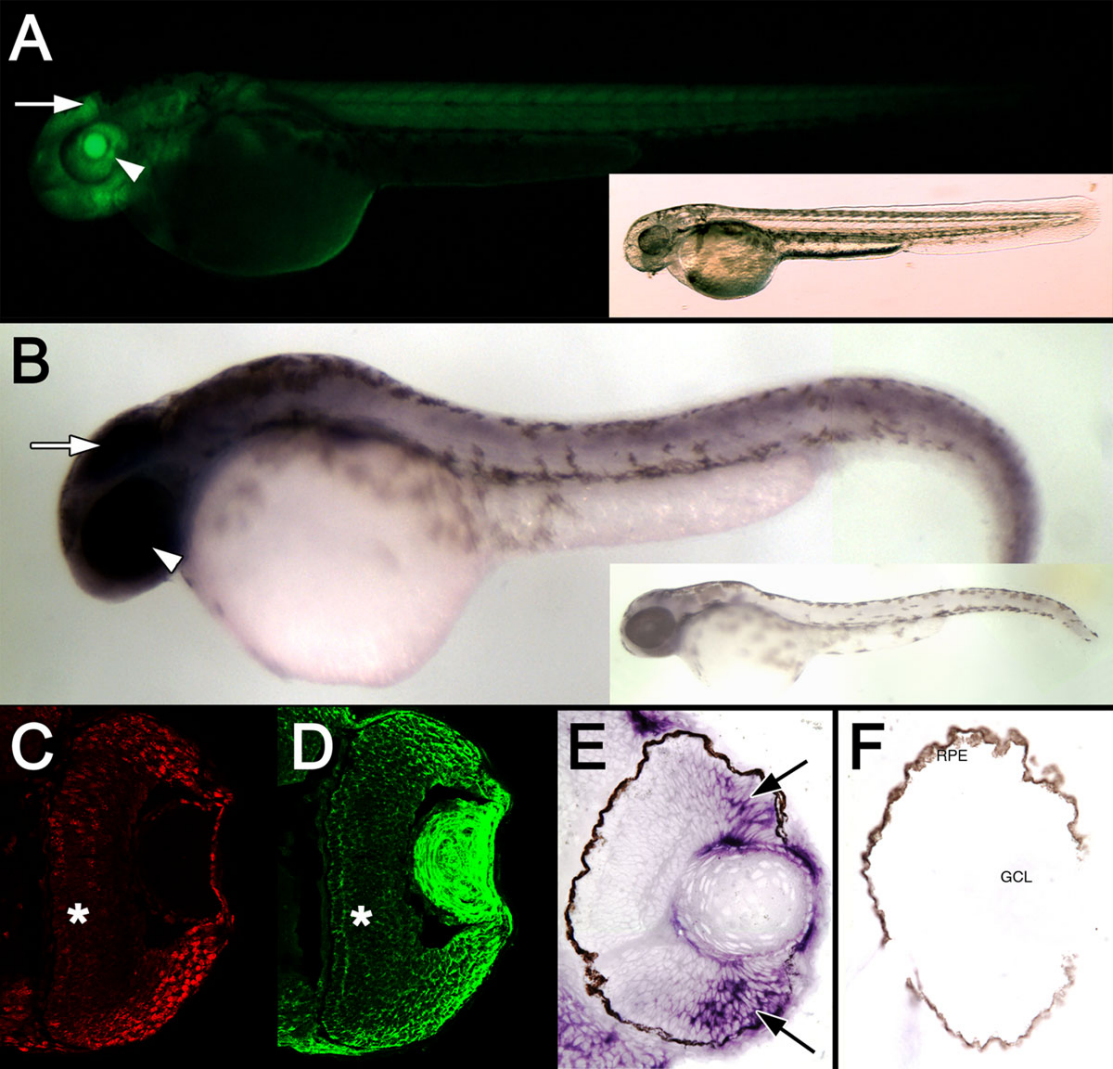
Expression of the *cyclin B1:EGFP* transgene at 48 hpf. **A**: A fluorescent image of a *Tg*(*ccnb1:EGFP*)^nt18^ embryo at 48 hpf is displayed. EGFP was expressed throughout the body of the fish with the most intense expression in the brain (arrow) and eye field (arrowhead). A bright-field image of the same embryo is shown in the inset. **B**: Whole-mount in situ hybridization revealed *cyclin B1* mRNA expression throughout the body with the greatest staining in the head (arrow) and eye (arrowhead). A zebrafish labeled with the *cyclin B1* sense RNA probe is shown in the inset. Retinal sections at 48 hpf reveal strong PCNA expression in cells in the retinal margin (**C**), which is similar to EGFP expression (**D**). EGFP and PCNA expression is decreased in the central retina (*). Retinal sections labeled with the *cyclin B1* antisense RNA probe demonstrated a similar high level of expression near the retinal margin (**E**, arrows), while the control sense RNA probe did not label any retinal cells. **F**: No signal was observed with the sense RNA probe. Abbreviations: RPE represents retinal pigmented epithelium, and GCL represents ganglion cell layer.

### Immunohistochemistry

Embryos and adult eyes were fixed in 9:1 ethanolic formaldehyde overnight at 4 °C. Embryos were cryoprotected in 5% sucrose/1X PBS overnight at 4 °C then in 30% sucrose/1X PBS overnight at 4 °C and finally in 30% sucrose/1X PBS:Tissue Freezing Media (1:1; Triangle Biomedical Sciences, Durham, NC) for 4 h at room temperature. Tissue was then embedded and frozen in 100% Tissue Freezing Medium and sectioned at 18 μm. Slides were dried at 50 °C for 2 h. Tissue sections were rehydrated with 1X PBS, blocked with 1X PBS/2.5% normal goat serum/0.3% Triton X-100/1% DMSO, and incubated overnight at 4 °C with a 1:1500 dilution of rabbit anti-EGFP polyclonal antiserum (Abcam, Cambridge, MA), a 1:1000 dilution of mouse anti-PCNA polyclonal antiserum (Sigma, St. Louis, MO), a 1:1000 dilution of mouse anti-glutamine synthetase monoclonal antiserum (Chemicon, Temecula, CA), a 1:1500 dilution of mouse anti-EGFP monoclonal antiserum (Abcam), or a 1:250 dilution of rabbit anti-Pax6 polyclonal antiserum (Covance, Berkely, CA). Sections were washed three times in 1X PBS/0.1% Tween-20. Goat anti-rabbit Alexa Fluor 488 or 594 and goat anti-mouse Alexa Fluor 488- or 594-conjugated secondary antibodies (Molecular Probes, Carlsbad, CA) were diluted 1:500 in the blocking buffer, and sections were incubated with the secondary antibody for 1 h at room temperature. Sections were washed three times in 1X PBS/0.1% Tween-20, and then mounted using Vectashield (Vector Laboratories, Burlingham, CA). The Bio-Rad 1024 confocal microscope (Bio-Rad, Hercules, CA) was used to take 10 micron Z-stacked images.

For Pax6 labeling, retinal sections were rehydrated in 1X PBS and then incubated in preheated 1X AntigenPlus buffer (pH 10; Novagen, Madison, WI) for 25 min at 95 °C. Slides were cooled for 45 min and immunolabeled as described above.

### In situ hybridization

A 550-bp segment of the zebrafish *cyclin B1* open reading frame was PCR-amplified (Forward: 5′-CGA GTC ACA GCA ATA AAC CAC GAG-3′ and Reverse: 5′-TTC CCA GTA ACT TCC TTT CCT GC-3′) and ligated into a pCR 4-TOPO vector (Invitrogen, Carlsbad, CA). The construct was linearized then in vitro transcribed with either a T3 or T7 RNA polymerase (Ambion, Austin, TX) and labeled with digoxigenin UTP (Ambion). Whole-mount in situ hybridization with sense or antisense digoxigenin-labeled RNA probe was performed as previously described [51] with minor modifications. Embryos were fixed in 4% paraformaldehyde/5% sucrose overnight at 4 °C. Following dehydration in methanol and a 20 min digestion with Proteinase K (10 μg/ml in 1X PBS/0.1% Tween-20), embryos were fixed in 4% paraformaldehyde/1X PBS and then incubated for 2 h at 55 °C in hybridization buffer (50% formamide, 5X SSC, 500 μg/ml yeast tRNA, 0.1% Tween-20, 1% CHAPS, and 100 μg/ml heparin). *Cyclin B1* digoxigenin-labeled sense and antisense RNA probes were incubated with embryos for three days at 55–60 °C. Embryos were washed, incubated for 2 h at room temperature in blocking solution, and then incubated overnight at 4 °C in blocking solution containing alkaline phosphatase-conjugated anti-digoxigenin Fab fragments (Roche, Indianapolis, IN) that had been pre-absorbed with embryos from the same stage. Following washes in staining buffer (100 mM Tris [pH 9.5], 50 mM MgCl_2_, 100 mM NaCl, 0.1% Tween-20, 1 mM levamisole), embryos were incubated in BM purple (Roche) substrate for 1.5 h at 37 °C for whole-mount imaging. For embryonic retinal sections, embryos were incubated in BM purple substrate for two days at 4 °C then fixed in 4% paraformaldehyde/1X PBS overnight at 4 °C. Embryos were embedded in JB-4 plastic resin (Polysciences, Warrington, PA) for two days at room temperature. Two and a half μm sections were cut and mounted on slides, and pictures were taken on a Nikon Microphot-FXA upright microscope with a RTKE Spot camera (Diagnostic Instruments Inc., Sterling Heights, MI).

**Figure 3:**
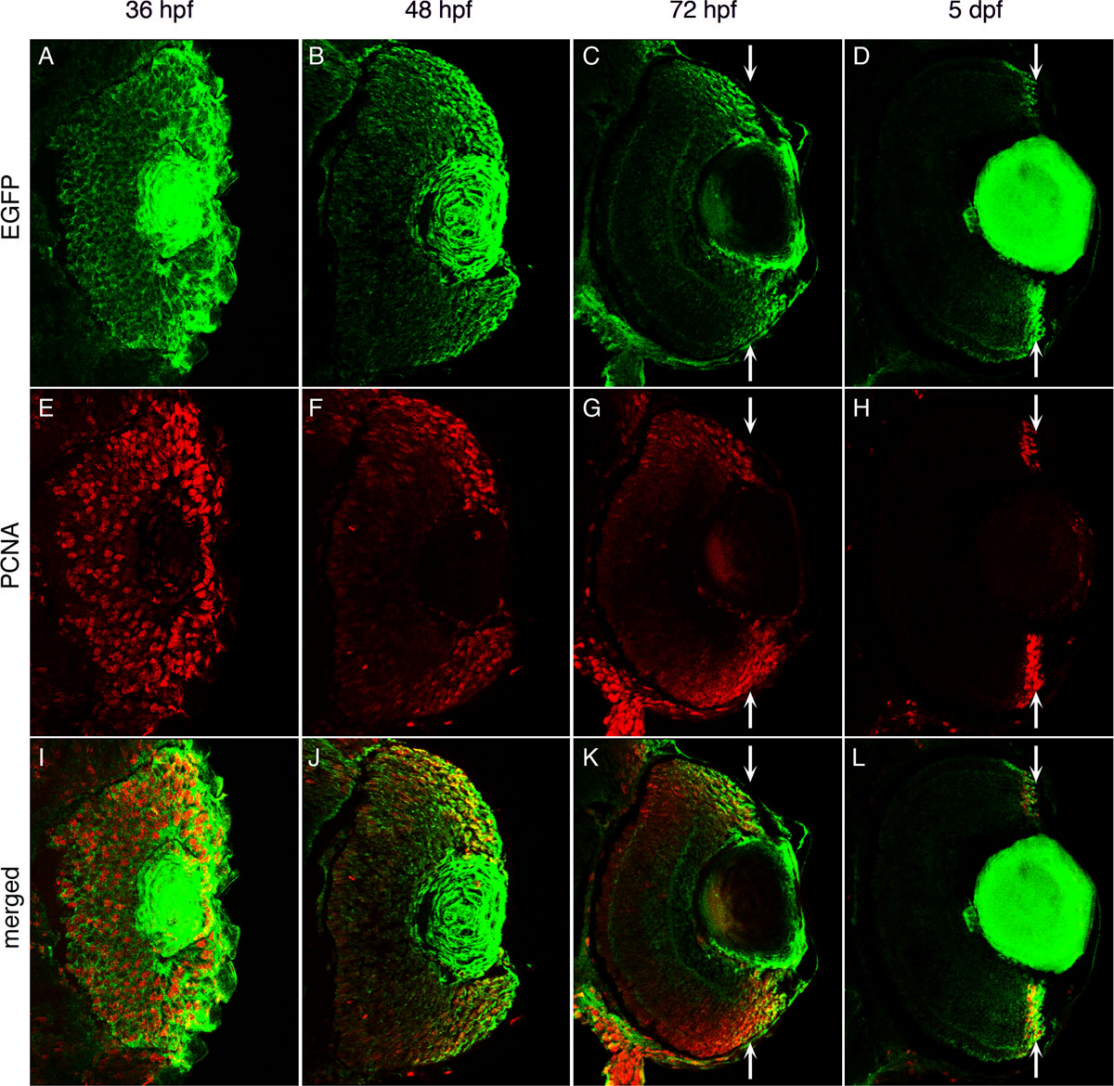
Enhanced green fluorescent protein expression during retinal development in *Tg(ccnb1:EGFP)*^nt18^ zebrafish. Enhanced green fluorescent protein (EGFP; **A-D**), proliferating cell nuclear antigen (PCNA; **E-H**), and merged expression patterns (**I-L**) are shown at 36, 48, 72 hpf, and 5 dpf of retinal development. At 36 hpf, PCNA and EGFP expression are almost ubiquitous throughout the retina (**A, E, I**). At 48 hpf, EGFP and PCNA expression is limited in the central retina, but persists near the retinal margin (**B, F, J**). At 72 hpf, EGFP and PCNA expression are largely absent in the central retina and become further focused in the cells near the margin (**C, G, K**; arrows). By 5 dpf, EGFP and PCNA expression are restricted to the retinal margin (D, H, L; arrows). At all time points, EGFP expression but not PCNA persists in the lens.

## Results

### Establishing a *cyclin B1:EGFP* transgenic zebrafish line

A 1.5 kb fragment of the *cyclin B1* promoter that is upstream of the translation initiation codon was PCR-amplified and cloned upstream of the *EGFP* reporter gene within the zebrafish Tol2 transposable element pT2KXIG plasmid (Figure 1). This construct was co-injected with in vitro transcribed Tol2 transposase mRNA into one to four-cell stage AB strain embryos. The EGFP-positive F0 fish were outcrossed to AB fish. The resulting EGFP-positive F1 carriers were identified and used to generate two independent *Tg*(*ccnb1:EGFP*)^nt18^ transgenic lines. Both transgenic lines had the same EGFP expression pattern in the retina during development, as an adult, and during retinal regeneration.

At 48 h post-fertilization (hpf), the *Tg*(*ccnb1:EGFP*)^nt18^ zebrafish head and eye exhibited strong EGFP expression, and the midsection and posterior of the fish expressed EGFP at a reduced intensity compared to the anterior of the fish (Figure 2A). This EGFP expression pattern was confirmed by whole-mount in situ hybridization with an in vitro transcribed *cyclin B1* antisense mRNA probe. At 48 hpf, the *cyclin B1* mRNA was strongly expressed throughout the head including the retina (Figure 2B). We also detected *cyclin B1* mRNA throughout the body and tail of the fish but at a reduced level relative to the anterior portion of the fish (Figure 2B). Thus, the EGFP expression throughout the 48 hpf *Tg*(*ccnb1:EGFP*)^nt18^ embryo appeared to mimic the expression of the endogenous *cyclin B1* mRNA.

**Figure 4:**
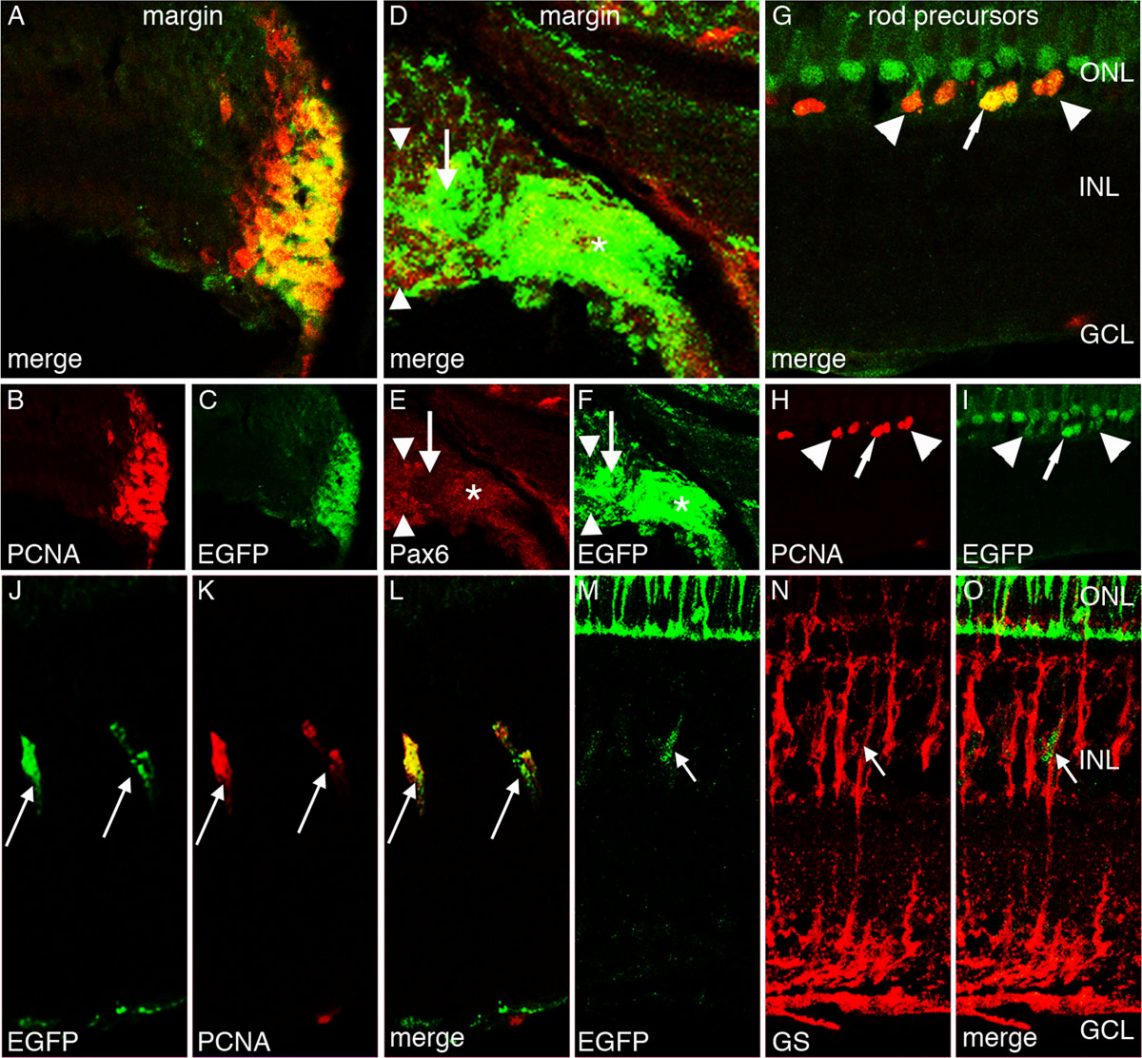
Enhanced green fluorescent protein expression in the *Tg*(*ccnb1:EGFP*)^nt18^ adult zebrafish retina. Enhanced green fluorescent protein (EGFP; **C, F, I, J, M**), proliferating cell nuclear antigen (PCNA; **B, H, K**), Pax6 (**E**), glutamine synthetase (GS; **N**), and merged expression (**A, D, G, L, O**) are shown. EGFP and PCNA are strongly expressed in the cells of the adult retinal circumferential marginal zone (CMZ; **A-C**). EGFP expression in the peripheral CMZ co-labels with Pax6 (*), but does not co-label near the distal CMZ (**D-F**, arrow). The Pax6-positive EGFP-negative signal adjacent to the CMZ (arrowheads) corresponds to the newly differentiated amacrine and ganglion cells. EGFP is also expressed in PCNA-positive rod precursor cells, which reside in the outer nuclear layer (**G-I**). Some of these rod precursor cells express high levels of EGFP (arrows), while others express lower EGFP levels (arrowhead). EGFP is also expressed in a row of nonproliferative PCNA-negative short single cones (**G, I**). Some Müller glia in the adult undamaged retina slowly divide and give rise to retinal progenitor cells. PCNA marks these proliferating Müller glial cells, which also coexpress EGFP (**J-L**, arrows). EGFP-positive cells in the INL also co-label with glutamine synthetase (**M-O**, arrow). This demonstrates that proliferating EGFP-positive cells co-label with Müller glia. Abbreviations: ONL represents outer nuclear layer; INL represents inner nuclear layer; and GCL represents ganglion cell layer.

### Enhanced green fluorescent protein expression during zebrafish retinal development

At 28 hpf, the entire zebrafish retina is composed of proliferating retinal neuroepithelial cells [52]. At this time, a group of cells in the ventronasal region of the retina exit the cell cycle and differentiate into ganglion cells [52]. Neurogenesis continues in a wave from the central retina to the periphery [28,53]. After development of the three retinal nuclear layers, proliferation continues in the circumferential germinal zone (CGZ; circumferential marginal zone [CMZ] in the adult) throughout the remaining life of the fish [23]. *Tg*(*ccnb1:EGFP*)^nt18^ zebrafish were collected at 36, 48, 72, and 120 hpf to visualize cell proliferation during retinal development. At 36 hpf, EGFP expression was observed throughout the retina with stronger expression near the periphery of the sheet of undifferentiated retina (Figure 3A). Proliferating cell nuclear antigen (PCNA) is a well documented marker for cell proliferation in the zebrafish retina [19,24,44]. At 36 hpf, PCNA was expressed throughout the undifferentiated retina with a higher degree of expression in the periphery (Figure 3E), which nicely overlaid the EGFP expression in the *Tg*(*ccnb1:EGFP*)^nt18^ embryonic retinas (Figure 3I). At 48 hpf, EGFP and PCNA expression decreased in the central retinal cells as these cells are the first to exit the cell cycle [28,52], but expression persisted in the CGZ (Figure 3B,F,J). These data confirmed that the *cyclin B1:EGFP* transgene was expressed in proliferating cells in the early stages of zebrafish retinal development.

EGFP was also expressed in the lens of *Tg*(*ccnb1:EGFP*)^nt18^ embryos at 36, 48, and 72 hpf, as well as at 5 days post-fertilization (Figure 3A-D). While the lens contains a population of proliferating lens epithelial cells, the EGFP expression is found throughout the lens. This unexpected broad expression pattern, which was not observed with PCNA expression, is either due to unusually stable EGFP expression or ectopic expression in all lens cell types. We found that EGFP was also misexpressed in the lens of three other transgenic lines that our laboratory either generated or examined, the rod photoreceptor cell *rho* promoter Tg(*rho:EGFP*) transgenic line [34], the progenitor cell *olig2* promoter *Tg*(*olig2:EGFP*)*^vu12^* transgenic line [45], and the Müller glial *gfap* promoter in *Tg(gfap:EGFP)^nt11^* [19,36]. The reason for the misexpression of several different transgenes in the lens remains unknown.

To further confirm that the transgene expression pattern recapitulated the endogenous *cyclin B1* expression pattern, we performed whole-mount in situ hybridizations with an in vitro transcribed *cyclin B1* mRNA probe and sectioned the labeled retinas. At 48 hpf, the *cyclin B1* mRNA transcripts were primarily detected near the CGZ with decreasing levels visualized in the central portion of the retina (Figure 2E). This pattern was similar to the EGFP and PCNA expression patterns in the *Tg*(*ccnb1:EGFP*)^nt18^ embryos at 48 hpf (Figure 2D,C, respectively). As retinal development proceeded to 72 hpf, EGFP and PCNA expressions became further restricted to the retinal margin (Figure 3C,G,K). By 5 dpf, EGFP expression was confined to the CMZ, which corresponded to the PCNA expression pattern (Figure 3D,H,L) where retinal cell proliferation persists throughout the lifetime of the zebrafish [23,35,54].

### Enhanced green fluorescent protein expression in the undamaged adult *Tg*(*ccnb1:EGFP*)^nt18^ zebrafish retina

The adult zebrafish retina contains three distinct sources of mitotically active cells, the CMZ, ONL rod precursor cells, and INL Müller glia [22,23,35,54]. We performed immunohistochemistry on the undamaged adult *Tg*(*ccnb1:EGFP*)^nt18^ retinal sections to determine if EGFP was expressed in these three progenitor cell populations. Similar to the 5 dpf retina, EGFP was highly expressed in the adult retinal CMZ (Figure 4C), which co-labeled with PCNA expression (Figure 4A,B). EGFP-positive cells near the periphery of the CMZ coexpressed Pax6 (Figure 4D-F), an early marker for neuronal progenitor cells in the CMZ [23], while the EGFP-positive cells toward the central CMZ had minimal Pax6 expression (Figure 4D-F, arrow). The further centrally located Pax6-positive EGFP-negative signal corresponds to the newly differentiated amacrine and ganglion cells (Figure 4D-F, arrowheads). Rod precursor cells in the ONL are specialized progenitors that are committed to the rod photoreceptor cell lineage during the persistent adult retinal neurogenesis [18,35]. EGFP and PCNA were coexpressed in these proliferating rod precursor cells (Figure 4G-I, identified by arrow). These rod precursor cells arise from a subset of Müller glia in the undamaged adult zebrafish retina [55], where a limited number of Müller glia reenter the cell cycle and produce neuronal progenitor cells that migrate to the ONL and become rod precursor cells during persistent neurogenesis [22,36,55]. In the *Tg*(*ccnb1:EGFP*)^nt18^ zebrafish retina, we identified a few glutamine synthetase (GS)-positive Müller glia that coexpressed PCNA and EGFP (Figure 4J-O). In the central retina, there was unexpected EGFP expression in PCNA-negative short single cone cells (Figure 4G,I). The EGFP expression in the short single cones may be either a failure to clone all the silencer elements associated with the *ccnb1* promoter or the actual representation of *cyclin B1* expression in the differentiated short single cones, similar to the expression of cyclins and cyclin-dependent kinases in other differentiated neurons [56]. Regardless, there was an excellent correspondence between EGFP expression and the three proliferating progenitor cell populations in the undamaged adult *Tg*(*ccnb1:EGFP*)^nt18^ retina.

**Figure 5:**
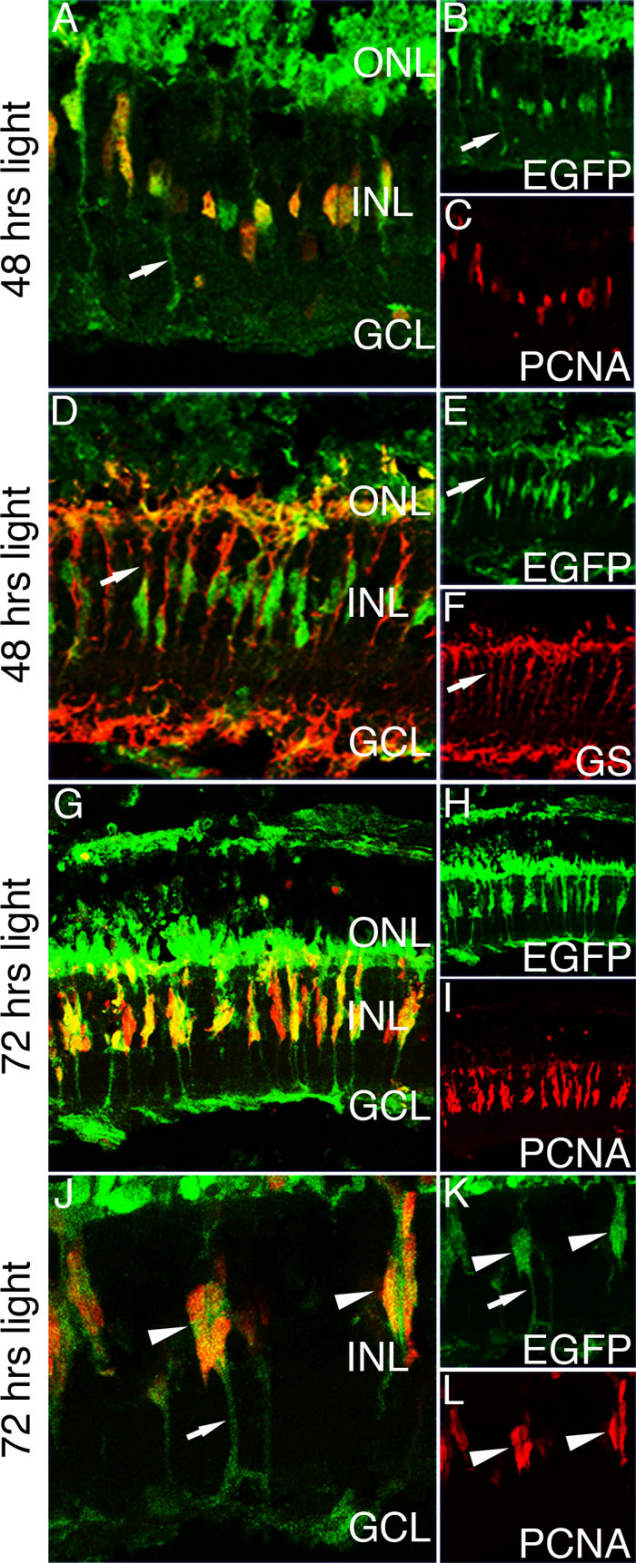
Enhanced green fluorescent protein expression in the light-damaged *Tg*(*ccnb1:EGFP*)^nt18^ zebrafish retina. To initiate the regeneration response, dark-adapted adult *Tg*(*ccnb1:EGFP*)^nt18^ zebrafish were exposed to constant, high-intensity light to induce photoreceptor apoptosis. At 48 h of constant light exposure, enhanced green fluorescent protein (EGFP) is expressed in proliferating cell nuclear antigen (PCNA)-positive inner nuclear layer (INL) cells (**A-C**). EGFP expression is also observed in Müller glial cell processes (arrows). At 48 h of light damage, Müller glia are labeled with glutamine synthetase (GS; **D, F**). All the EGFP-positive cells co-label with glutamine synthetase expressing Müller glia (**D-F**). At 72 h of light damage, there are clusters of PCNA-positive neuronal progenitor cells associated with and migrating along Müller glia (**G-I**). These cells express EGFP at a high intensity. (**J-L**) A higher magnification of the migrating progenitor cells at 72 h of light damage reveals that EGFP is expressed throughout the glial cell (**J, K**). This is evident by labeling in the glial cell processes (arrows). There are multiple PCNA-positive progenitors that are also expressing EGFP (arrowheads). Abbreviations: GS represents glutamine synthetase; PCNA represents proliferating cell nuclear antigen; EGFP represents enhanced green fluorescent protein; ONL represents outer nuclear layer; INL represents inner nuclear layer; and GCL represents ganglion cell layer.

### Enhanced green fluorescent protein expression in the regenerating *Tg*(*ccnb1:EGFP*)^nt18^ zebrafish retina

Damage to any cell type of the adult zebrafish retina will initiate a robust regenerative response [19,24-26,54,57]. Previously, our laboratory characterized a light damage model that destroys both rod and cone photoreceptors [19,21]. Photoreceptor cell death initiates a regenerative response with the Müller glia exhibiting an increased amount of proliferation and a generation of large clusters of neuronal progenitor cells, which migrate to the ONL and replenish the lost rod and cone photoreceptors [19,21]. To determine if the proliferating Müller glia in the regenerating retina expressed the *cyclinB1:EGFP* transgene, *Tg*(*ccnb1:EGFP*)^nt18^ zebrafish were exposed to constant light. After 48 h of constant light, EGFP was expressed in all the PCNA-expressing Müller glia (Figure 5A-C). Glutamine synthetase, a known marker for Müller glia [19,21], confirmed that the EGFP-positive cells in the light-damaged retina were Müller glial cells (Figure 5D-F). After 72 h of light damage, multiple progenitors were visualized migrating along the processes of the EGFP-positive Müller glia (Figure 5G-L). Therefore, EGFP expression in the *Tg*(*ccnb1:EGFP*)^nt18^ zebrafish line detected the proliferating Müller glia and neuronal progenitor cells during zebrafish retinal regeneration.

### Comparison of enhanced green fluorescent protein expression in the *Tg(1016α1tubulin:EGFP)* and *Tg*(*ccnb1:EGFP*)^nt18^ transgenic fish lines

**Figure 6:**
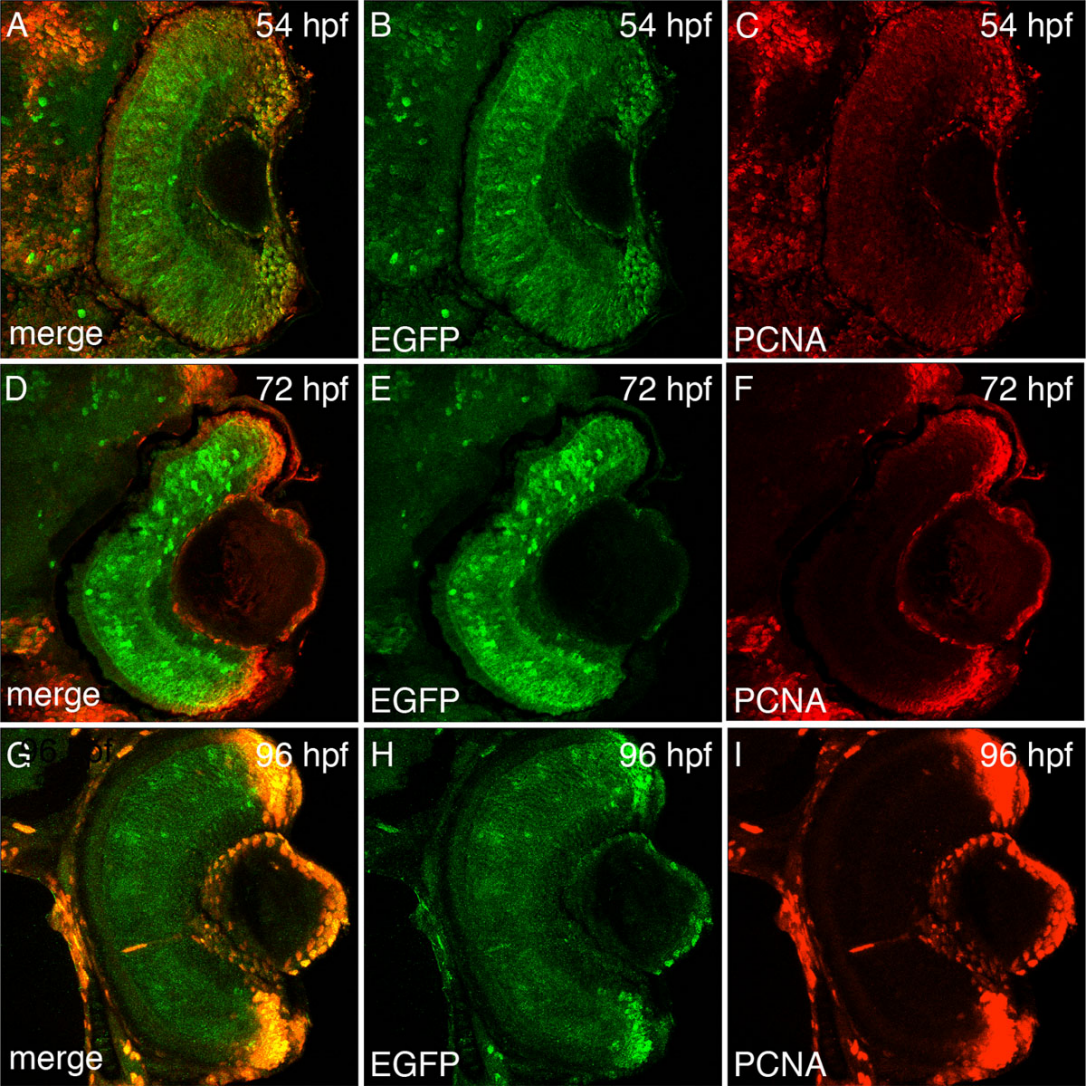
Enhanced green fluorescent protein expression during retinal development in *Tg(1016α1tubulin:EGFP)* zebrafish. Enhanced green fluorescent protein (EGFP; **B, E, H**), proliferating cell nuclear antigen (PCNA; **C, F, I**), and merged expression patterns (**A, D, G**) are shown at 54 hpf, 72 hpf, and 96 hpf of retinal development. At 54 hpf, EGFP is expressed throughout the entire retina, while PCNA expression is lower in the central retina relative to the retinal margin (**A-C**). At 72 hpf, EGFP continues to be expressed throughout the central retina, while PCNA expression is largely absent in the central retina and highly expressed in the cells near the margin (**D-F**). By 96 hpf, EGFP expression decreases in the central retina but remains in the margin, while PCNA expression remains restricted to the retinal margin (**G-I**).

EGFP expression in the *Tg(1016α1tubulin:EGFP)* zebrafish line, which contains a fragment of the *α1tubulin* promoter, co-labeled with proliferating Müller glia and neuronal progenitor cells during zebrafish retinal regeneration [22], which is similar to the *ccnb1:EGFP* transgene expression. We compared the EGFP expression profiles in the *Tg*(*ccnb1:EGFP*)^nt18^ transgenic line relative to the *Tg(1016α1tubulin:EGFP)* line during retinal development. At 54 hpf, EGFP in the *Tg(1016α1tubulin:EGFP)* zebrafish line was expressed throughout the retina while PCNA expression was mostly restricted to the retinal margin (Figure 6A-C). At 72 hpf, EGFP persisted in the central retina and PCNA was further restricted to the margin (Figure 6D-F). This differed from the early time points analyzed in the *Tg*(*ccnb1:EGFP*)^nt18^ transgenic line where EGFP decreased in the central retina relative to the margin (Figure 3B,F,J,C,G,K compared to Figure 6A-F). The EGFP expression in the *Tg(1016α1tubulin:EGFP)* transgenic line did not decrease in the central retina and become more restricted to the margin until 4 dpf (Figure 6G-I).

**Figure 7:**
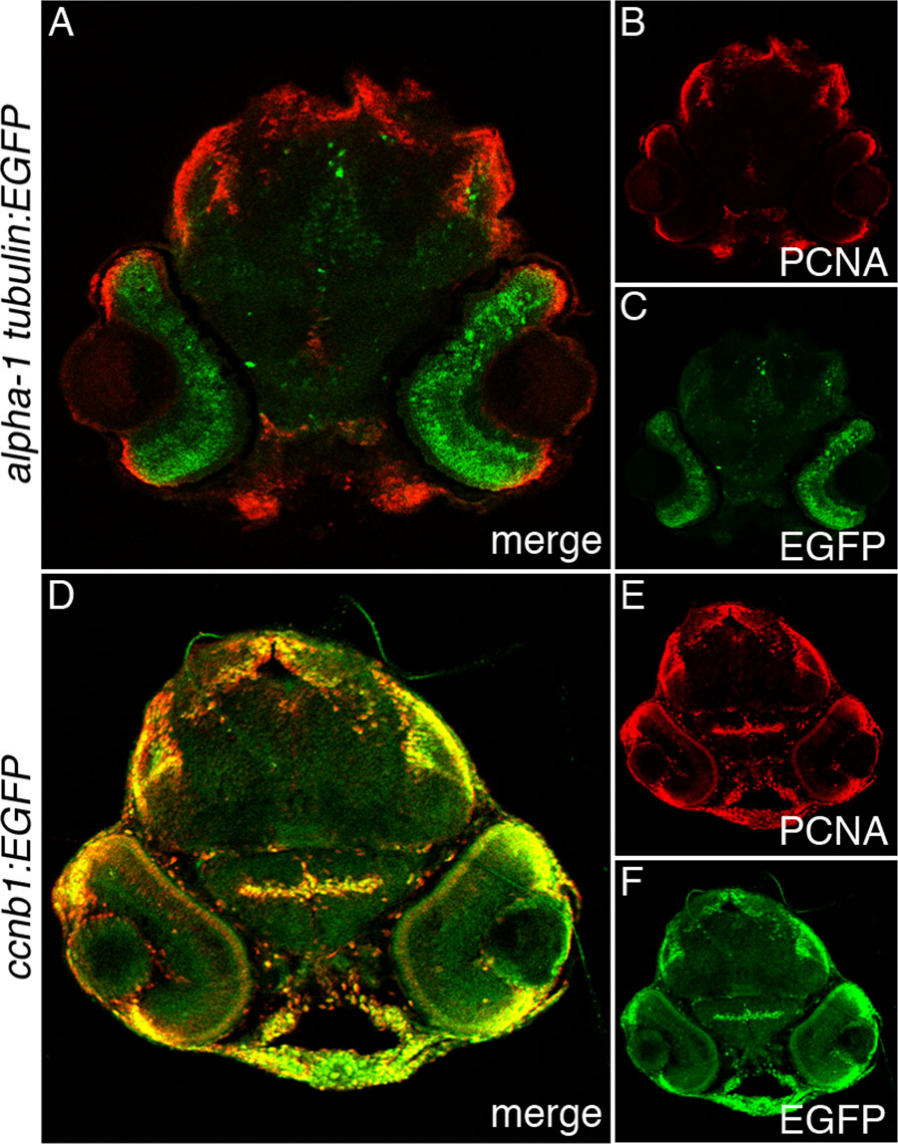
Enhanced green fluorescent protein and proliferating cell nuclear antigen expression in *Tg(1016α1tubulin:EGFP)* and *Tg*(*ccnb1:EGFP*)^nt18^ 72 hpf larva head sections. Enhanced green fluorescent protein (EGFP; **C, F**), proliferating cell nuclear antigen (PCNA; **B, E**), and merged expression patterns (**A, D**) are shown in 72 hpf larval head sections. In the *Tg(1016α1tubulin:EGFP)* zebrafish, EGFP was predominantly expressed throughout the retina, while PCNA was expressed in the retinal margin, mandibular tissue, the dorsal epithelium, and tectum (**A-C**). In the *Tg*(*ccnb1:EGFP*)^nt18^ zebrafish, EGFP was restricted to the retinal margins similar to PCNA, but EGFP was also expressed in other head tissues that coexpressed PCNA (**D-F**).

To further demonstrate the differences between the EGFP expression patterns in the *Tg*(*ccnb1:EGFP*)^nt18^ and *Tg(1016α1tubulin:EGFP)* lines, we examined EGFP and PCNA expression in the larval brain and retinal sections at 72 hpf. PCNA-expressing cells in the retinal margins, mandibular tissue, and dorsal epithelium and tectum did not co-label with EGFP in the *Tg(1016α1tubulin:EGFP)* transgenic line (Figure 7A-C). However, PCNA expression in similar regions of the *Tg*(*ccnb1:EGFP*)^nt18^ transgenic zebrafish line co-labeled with EGFP (Figure 7D-F).

## Discussion

To label the proliferating stem and progenitor cell populations in the developing, adult, and regenerating zebrafish retina, we generated the *Tg*(*ccnb1:EGFP*)^nt18^ transgenic line that expresses EGFP from a 1.5 kb *cyclin B1* promoter (*ccnb1*). We confirmed that the EGFP expression pattern in this transgenic line corresponded to proliferating cells by co-labeling with PCNA, a known marker for DNA replication, as well as in situ hybridization with a *cyclin B1* specific antisense RNA probe (Figure 2). In early retinal development, proliferating progenitors in the sheet of neuroepithelial cells expressed the *cyclin B1:EGFP* transgene. At 48 hpf, EGFP and PCNA expression was reduced in the central retina (Figure 3) as these cells began to exit the cell cycle and differentiate. By 5 dpf, EGFP and PCNA expression was restricted to the retinal margin (Figure 3), a source of persistently proliferating stem and progenitor cells throughout the life of the fish. In the adult retina, the *cyclin B1:EGFP* transgene continued to be expressed with PCNA in the retinal margin (CMZ), in proliferating rod precursor cells, and in a subset of Müller glia in both the undamaged and damaged retina (Figures 4 and 5).

Other transgenic lines label neuronal progenitor cells in zebrafish, such as the *Tg(1016α1tubulin:EGFP)* [22] and the *Tg*(*olig2:EGFP*)*^vu12^* [45] lines. We previously demonstrated in the regenerating retina that the *olig2:EGFP* transgene is not expressed in Müller glial cells when they begin proliferating [19,24]. In contrast, the *ccnb1:EGFP* transgene is first expressed in the Müller glia at the same time as PCNA expression (Figure 5A-C). We also performed a comparison of EGFP expression between the *Tg(1016α1tubulin:EGFP)* and *Tg*(*ccnb1:EGFP*)^nt18^ lines during development. EGFP expression in the *Tg*(*ccnb1:EGFP*)^nt18^ transgenic line mimicked the pattern of PCNA expression quite well at different stages of retinal development (Figure 3). EGFP expression in the *Tg(1016α1tubulin:EGFP)* transgenic line was present in the neuronal progenitor cells during retinal development (Figure 6). However, as PCNA expression became more restricted to the retinal margins between 54 and 72 hpf, EGFP expression remained throughout the central retina (Figure 6). Also, EGFP expression in the *Tg(1016α1tubulin:EGFP)* transgenic line did not always coexpress with PCNA in head sections at 72 hpf, while EGFP in the *Tg*(*ccnb1:EGFP*)^nt18^ transgenic line nearly always coexpressed with PCNA throughout the developing head at 72 hpf (Figure 7).

The *cyclin B1:EGFP* transgene was upregulated in proliferating Müller glial cells during zebrafish retinal regeneration. Multiple damage models had shown that the source of neuronal progenitor cells in the regenerating retina was an INL stem cell population that yielded rod precursor cells during persistent retinal growth [19,23,24,35,57]. Recently, many laboratories argued that this source of neuronal progenitors are Müller glia [21-23,36,57]. We showed that upon photoreceptor damage, Müller glia will reenter the cell cycle, dedifferentiate, and produce multiple progenitors that will use the glia as a scaffold to migrate to the ONL where they will replenish these lost rod and cone photoreceptors [19,21]. To further support the argument that an increased number of Müller glia proliferate during retinal regeneration, we showed that the *ccnb1:EGFP* transgene was expressed in a greater number of Müller glia in the light-damaged retina (Figure 5). Coimmunolocalization of EGFP and glutamine synthetase demonstrated that the *cyclin B1:EGFP* transgene was expressed in Müller glia (Figure 5D). Further, EGFP-positive Müller glia coexpressed PCNA (Figure 5A,G,J). Together, these data support the previous argument that an increased number of Müller glia reenter the cell cycle during regeneration of the damaged retina.

The *Tg*(*ccnb1:EGFP*)^nt18^ zebrafish can be used as a tool to study retinal development, retinal regeneration, and retinal diseases. In zebrafish retinal development, multipotent retinal progenitor cells in the neuroepithelial sheet exit the cell cycle, become neurogenic progenitor cells, and differentiate into all the retinal cell types that form the correct retinal laminar pattern by 3 dpf [27,28,58]. Various forward genetic screens and morpholino knockdown techniques revealed some of the mechanisms and proteins involved in retinal development [7,59]. There are multiple ways to screen for eye mutants, which include gross morphological phenotypes such as the size or absence of an eye, or for retinal behavior [7,32,60,61]. More detailed characterization can involve immunohistochemical labeling, in situ hybridizations, cell apoptosis assays, and electrophysiology techniques [62]. The screening of transgenic zebrafish for retinal mutants is potentially beneficial because it can reveal the temporal and spatial expression of specific cell types during development in both a live retina and retinal tissue sections. The *Tg*(*ccnb1:EGFP*)^nt18^ zebrafish was created to visualize proliferating cells in the developing retina in vivo relative to other markers such as PCNA and BrdU incorporation. PCNA and BrdU are not ideal for a genetic screen because they require fixation and immunolabeling, which can take several days to perform. However, observing whole-mount EGFP expression in the *Tg*(*ccnb1:EGFP*)^nt18^ zebrafish will readily permit the identification of developmental mutants, which can not properly enter or exit the cell cycle.
